# Features of p53 protein distribution in the corneal epithelium and corneal tear film

**DOI:** 10.1038/s41598-020-67206-z

**Published:** 2020-06-22

**Authors:** Yevgeny Tendler, Alexander Panshin

**Affiliations:** 1 Department of Clinical Biochemistry, Rambam Medical Center, Haifa, Israel; 2 Kimron Veterinary Institute, Beit Dagan, Israel

**Keywords:** Cancer, Cell biology

## Abstract

Tumor suppressor protein p53 is the key factor in the regulation of cell proliferation. Its concentration is low in the cytoplasm of most cell types. However, in corneal epithelium cells, abnormally high p53 content is detected. The aim of the present study was to characterize p53 distribution in the corneal epithelium. For this purpose, immunohistochemistry, western blot analysis and electronic microscope examinations were performed. A low level of p53 was identified in the lens, iris and retina; by contrast, a significantly high concentration of this protein was observed in the corneal epithelium. In opposite, MDM2 was identified in the lens, iris and retina while it is completely absent in the corneal epithelium. In addition, we found a significant amount of exosomes and other microvesicles containing p53 in the corneal mucin layer. We thus hypothesize that a significantly high level of p53 was caused by a combination of absents of MDM2 in parallel with p53 microvesicles storage.

## Introduction

Tumor suppressor protein p53, as a rule, is present in trace amounts in the vast majority of cells of adult organisms^1,2^ although a high synthesis of this protein is detected in certain tissues^3^. This is explained by the high rate of degradation of p53 in cytoplasmic proteasomes^1^. At the same time, a high level of p53 is detected in the cytoplasm of normal corneal epithelium cells of various vertebrate species^4^. Surprisingly, this protein, while present in significant quantities in the cytoplasm, does not exhibit its characteristic functional activities in unstressed normal conditions^5^.

The aim of the present study was to characterize the features of p53 protein distribution in the corneal epithelium and corneal tear film and to examine the possible factors that contribute to a significant accumulation of this protein in the cytoplasm of epithelial cells of the cornea.

## Materials and methods

### Animals

C57BL/6 mice and Sprague-Dawley rats were obtained from the Animal Facility of Technion (Haifa). The eyes of the animals were enucleated following sacrifice by CO_2_ narcosis. The use of animals adhered to the ethical guidelines of and was supervised by the Technion Animal Welfare Committee.

The objectives of the present study were histological preparations of the cornea. To examine the localization of p53, methods of immunochemical analysis (mouse), including immune electron microscopy (rat) of colloidal gold were used. The techniques of immunochemical analysis have been previously described in detail^4^. To examine the availability of the protein MDM2, the main antagonist of p53, in the corneal epithelium of rat, western blot analysis with classical modifications was used^6^. Equal amounts of protein derived from the respective ocular tissues were compared to each other by western blot analysis, following resolution of the protein by standard denaturation by 7.5% SDS polyacrylamide gel electrophoresis using standard conditions^6^. Proteins were transferred onto nitrocellulose membranes (Schleicher & Schuell), as previously described^7^. Bovine serum albumin (BSA)-blocked western blots were subjected to western blot analysis using the MABs 248 (a kind gift from Professor V. Rotter) and MDM2 (Clone SMP14) (Santa Cruz Biotechnology) followed by HRP-conjugated anti-mouse IgG (ECL Amersham Biosciences) at a dilution of 1:1,000 for 1 h at room temperature (RT). Between incubations, the blots were subjected to enhanced chemiluminescence (ECL) substrates to develop the results (Amersham Biosciences). ‘Sea-Blue protein molecular weight markers’ (NOVARIS) were used on each gel. Fifty micrograms of^8^ p53-M clone 314 cell extract (a kind gift from Professor V. Rotter, Weizman Institute, Rehovot, Israel) was used as a p53-positive control.

For the study of microvesicles from the aqueous mucin layer, a specific method was used. Microvesicles from the aqueous mucin layer of rats loaded onto formvar carbon-coated grids (Ted Pella Inc.) by the direct application of the grids to the conjunctival sac. P53 expression was mapped by indirect immunohistochemical staining techniques using the anti-p53 monoclonal antibodies (a kind gift from Professor V. Rotter), PAb 421 and PAb 248 (alternatively known as mAb 421and mAb 248, respectively).

### Visualization of microvesicles by electron microscopy

Microvesicles from the aqueous mucin layer of rats were loaded onto formvar carbon-coated grids (Ted Pella Inc.) by direct application of the grids to the conjunctival sac. The microvesicles on the grids were then fixed in 2% paraformaldehyde and washed and subsequently immunostained with mouse anti-p53 antibody Mab 421, Mab 248 or mouse monoclonal antibodies anti-CD63 (clone AD1) from BD Biosciences (Mountain View, CA, USA) followed by staining with a 10 nm gold-labelled anti-mouse secondary antibody (Sigma-Aldrich). The grids were subsequently fixed in 2.5% glutaraldehyde, washed, contrasted in 2% uranyl acetate and embedded in a mixture of uranyl acetate (0.8%) and methyl cellulose (0.13%). The preparations were examined under a JEOL JEM-1011 transmission electron microscope.

### Ethics approval and consent to participate

The use of animals adhered to the ethical guidelines of and was supervised by the Technion Animal Welfare Committee. The animals were maintained in conformity with the Guiding Principles in the Care and Use of Animals of the American Physiological Society, and the experimental protocols were approved by the Animal Welfare and Ethics Committee of the Technion-Faculty of Medicine.

## Results

### p53 expression in the cornea

**Figure 1:**
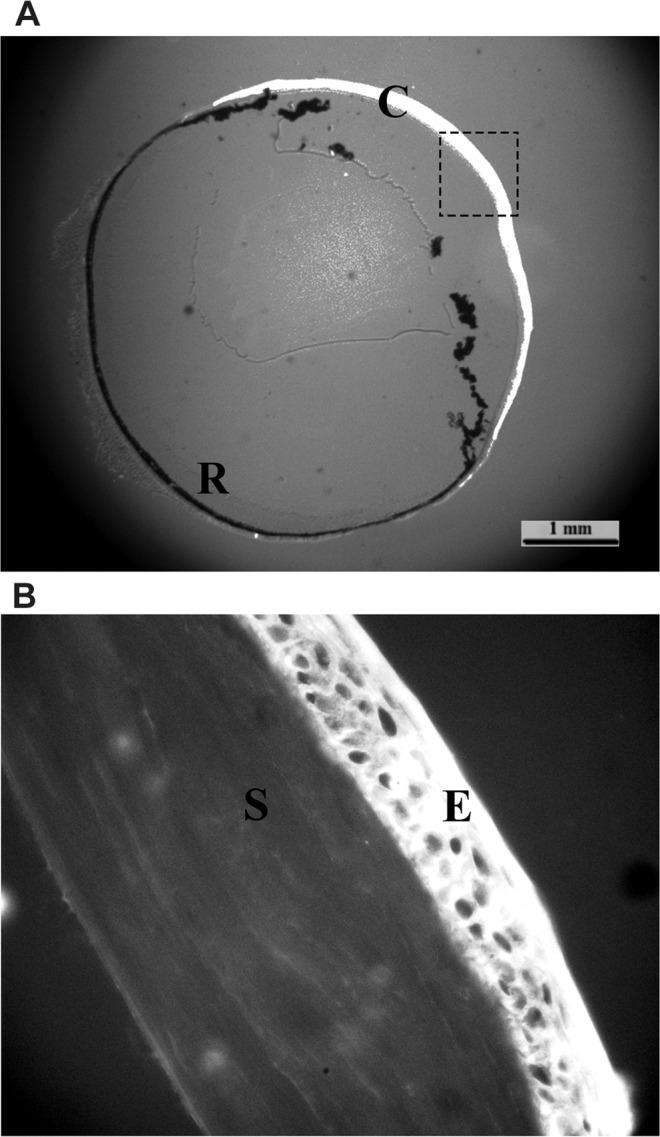
p53 protein in the mouse (C57Bl/6) eye. (**A**) The whole eye was observed using mAb 421 (magnification, x40) with positive staining of the corneal epithelium; C, cornea; R, retina. (**B**) Positive staining of corneal epithelium (magnification, x400); E, corneal epithelium; S, stroma.

Positive p53 staining was observed with mAb 421 in the cytoplasm of conjunctival and corneal epithelium cells, while endothelial cells and the stroma produced negative staining (Fig. 1A,B).

Since p53 often presents in cells in complex with its main antagonist MDM2, known as E3 ubiquitin-protein ligase, the distribution of this protein in the eye tissues was examined.

**Figure 2:**
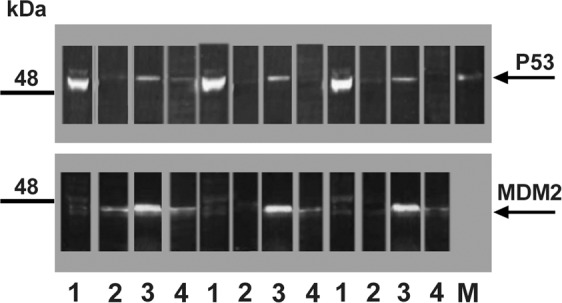
Western blot analysis of the normal rat ocular tissues; 1, cornea; 2, iris; 3, lens; 4, retina; M, p53 positive control. Repeated designations 1, 2, 3, 4 are replicates of the same tissues. Western blot were subjected to western blot analysis using the MABs 248 and MDM2 (Clone SMP14) followed by HRP-conjugated anti-mouse IgG. See also, whole membrane image as supplementary Fig. S1.

Using western blot analysis (Fig. 2, see also in Fig. S1, Supplement) significant amount of MDM2 48-kDa isoform^9,10^ was detected in the lens, iris and retina, while it was undetectable in the corneal epithelium.

**Figure 3:**
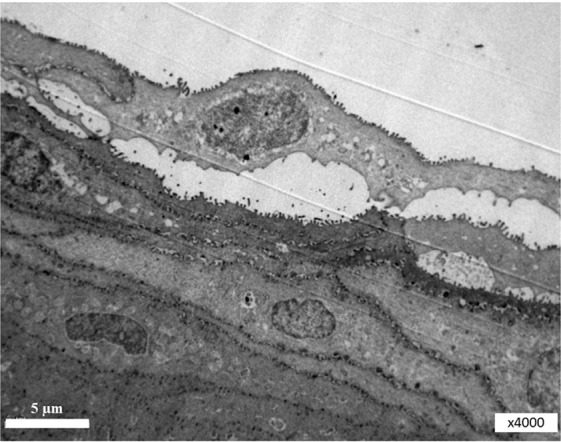
Surface of the corneal epithelial cell. Microvilli from the grey surface of the cornea.

**Figure 4:**
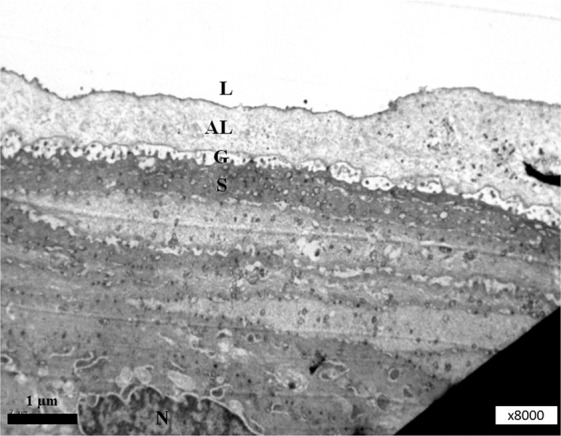
Surface of the mouse corneal epithelial cell with corneal tear film. L, lipid layer; AL, aqueous layer; G, glycocalyx; S, surface of the corneal epithelial cell; N, nucleus. Magnification, x 8k.

**Figure 5:**
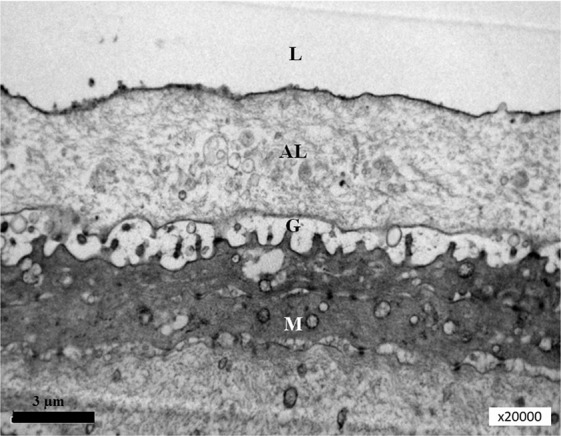
L, lipid layer; AL, aqueous layer (contains soluble mucins and microvesicles); G, glycocalyx (microvilli from the surface of the corneal epithelial cell); M, microvesicles in the cytoplasm of the corneal epithelial cell. Magnification, x20k.

Cells secrete membrane-derived vesicles, such as exosomes, ectosomes, shed vesicles, or microvesicles. Which bud directly from the cell’s plasma membrane. Microvilli from the surface of the corneal epithelial cells are very similar to microvesicles, also budding directly from the plasma membrane. These vesicles are known to carry active proteins and RNAs (Fig. 3). A significant amount of microvesicles was found in the conjunctival mucin layer (Figs. 4 and 5).

**Figure 6:**
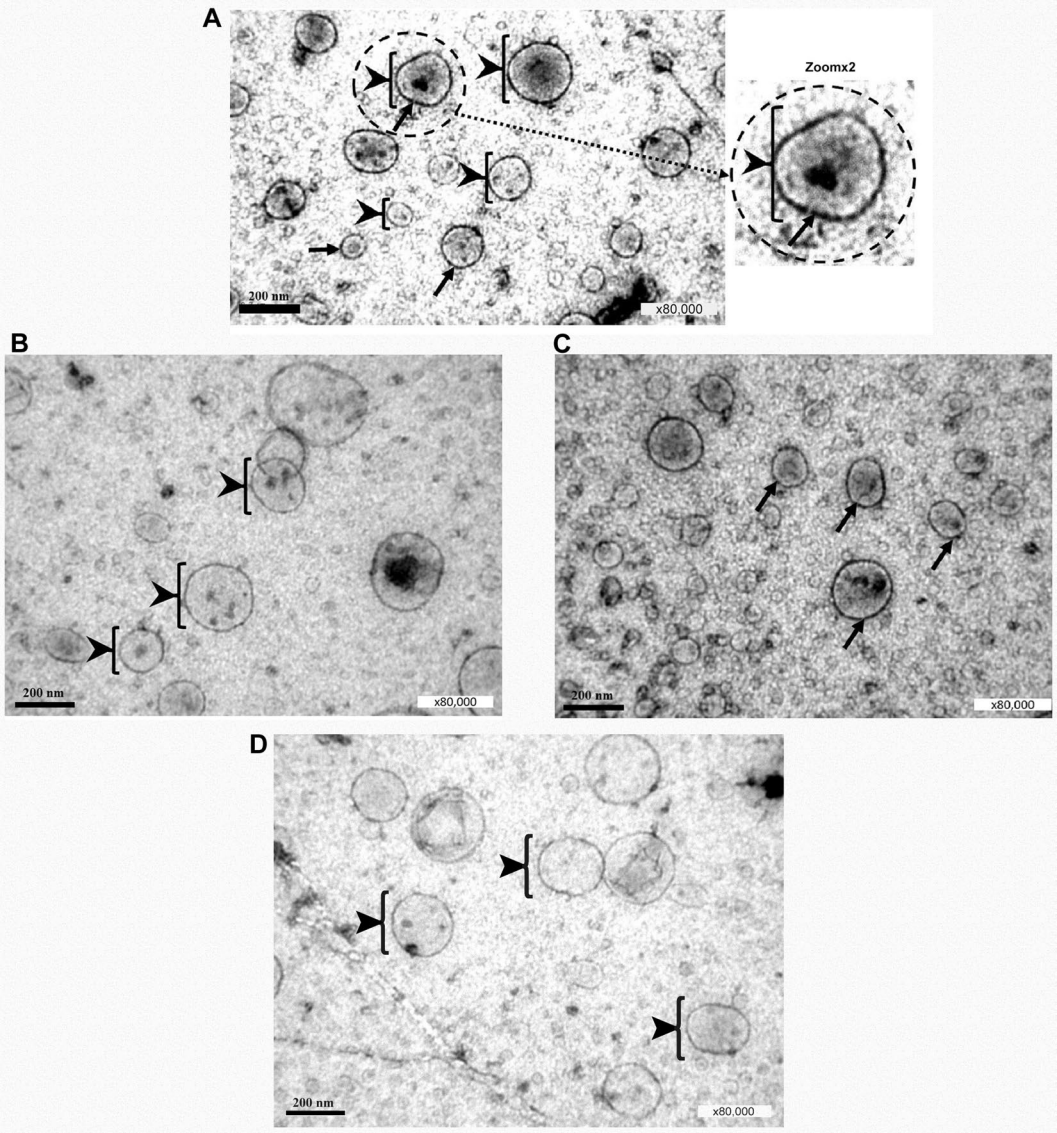
Electron micrograph of exosomes in the conjunctival mucin layer of rats. (**A**) Electron micrograph of p53 containing exosomes in the tear film of rats. Black arrow heads, exosomes; D = 50-200 nm. Dot dash arrows, gold particles D = 10 nm. The primary antibody used was Mab 421. The secondary antibody used was anti-mouse IgG-Gold. Magnification, x80K. (**B**) The primary antibody used was anti-p53 Mab 248. The secondary antibody used was anti-mouse IgG-Gold. p53 protein in the exosomes was not observed using mAb 248. **No gold particles**. Black arrow heads denote exosomes of 50-200 nm in size. Magnification, x80k. (**C**) Electron micrograph of CD63-positive exosomes in the tear film of rats. The primary antibody used was the exosome marker anti-CD63. The secondary antibody used was anti-mouse IgG- Gold. Solid arrows denote exosomes with gold particles. (**D**) Electron micrograph without the first antibody. No gold particles were observed with only anti-mouse IgG-Gold as a control for back-ground staining.

p53-containing microvesicles were present in the aqueous layer of corneal (preocular) tear film. Significant amounts of these exosomes were found using an electronic microscope in the aqueous mucin layer of rats (Fig. 6A–D). P53 protein in the exosomes was observed using mAb 421 (Fig. 6A), but not mAb 248. No gold particles were observed with mAb 248 (Fig. 6B). This may prove the specificity of positive staining with mAb 421 due to the lack of binding of mAb 248 to non-denatured p53^4,5^. The positive staining of some microvesicles with the exosome marker CD63 may prove that these are indeed exosomes (Fig. 6C). No gold particles were observed with only anti-mouse IgG-Gold as a control for back-ground staining (Fig. 6D).

Recently, different microvesicles and exosomes have been found in tear film and corneal epithelium cells^11,12^.

## Discussion

It has been shown that the unstressed normal corneal epithelium of adult vertebrates is characterized, in contrast to other tissues, by high levels of p53 expression^4^.

The p53 gene is a well-defined tumor-suppressor gene. The gene produces a 53-kDa phosphoprotein that was first characterized as the major cellular protein associated with the T antigen encoded by simian virus 40 (SV40), a small DNA virus^13^.

In general, the target genes of p53 can be grouped into categories of biological activities that include apoptosis, senescence, growth arrest, DNA repair, antioxidant defense and metabolic homeostasis. In addition to its role as a tumor suppressor by triggering apoptosis, cell cycle arrest and senescence, p53 also regulates the synthesis and secretion of exosomes from the stressed cells^14^. Under normal conditions, the protein levels of the p53 tumor suppressor are largely regulated by its interaction with MDM2^1,3^.

MDM2, which is the major E3 ubiquitin ligase of p53, can bind to the p53 tumor-suppressor protein and negatively regulate its function^15^. P53 normally regulates its own inactivation, by inducing MDM2 expression. The down-regulation of p53 by MDM2 is of utmost importance during embryonic development, since the lethality resulting from the inactivation of the MDM2 gene in mice is reversed by the inactivation of the p53 gene^16^. MDM2 forms a complex with p53, inhibiting its ability to stimulate transcription^17^ and stimulates its degradation via the ubiquitin pathway^18,19^. Usually MDM2 mediates p53 degradation and maintains low or undetectable levels of p53 in unstressed normal (non-malignant) cells^20^. The exception is the corneal epithelium. MDM2 expression was significant in the lens, iris and retina, while it was completely absent in the corneal epithelium. Similarly, only weak MDM2 expression was observed in conjunctiva tissues^21^ which is adjacent to the corneal epithelium.

A significant amount of p53 has been consistently detected in the cytoplasm of corneal epithelial cells by different methods^4,5,7,22–24^.

Previous studies have demonstrated p53 expression in the normal murine and rat corneal epithelium^7,22^ and have further mapped and characterized p53 expression in the normal cornea across different species^4^. Immunohistochemistry and western blot analyses have revealed a strong cytoplasmic p53 expression in the corneal epithelium of various vertebrate species. The observed corneal epithelial staining was localized in the cytoplasm and was absent in the cell nuclei.

Cytoplasmic p53 expression has been found to be positive in the corneal epithelium of the rat, mouse, bovine, chicken, gecko and *Hyla arborea* (tree frog), and to be negative in *Drosophila*. In *Drosophila*, no p53 staining was observed with any antiserum^4^.

Since MDM2 is the major E3 ubiquitin ligase of p53, its absence may be a reason for the ineffective ubiquitination and accumulation of p53 protein in the corneal epithelium. The combination of active p53 transcription with the absence of the p53 ubiquitin ligase MDM2 seems to account for the abundant presence of p53 in the cytoplasm of the corneal epithelium. Vice versa, recent research indicates high expression of MDM2 in pterygium tissues and hypothesize that enhanced p53 inhibition by MDM2 could play a role in the occurrence of pterygium^21,25^. In similar way, we hypothesize that MDM2 absents in corneal epithelium enables high p53 stability, enhance its concentration and leads to local anti-cancer defense.

In addition to its role as a tumor suppressor, p53 also regulates the synthesis and secretion of exosomes. The role of p53 as the “master exosome secretor” was shown by^14^.

Exosomes are small membrane vesicles (40-200 nm) first discovered 30 years ago^26^. Exosomes have pleiotropic biological functions, including immune response, antigen presentation, intracellular communication and the cells to cells transfer of RNA and proteins^27^. Cells also produced other membrane-derived microvesicles, which bud directly from the cell’s plasma membrane. These microvesicles are also known to carry active proteins and RNAs^27^. It was recently indicated that p53 protein can be transferred by exosomes from cell to cell and may play an important physiological role^28^. Exosomes are actually signaling payloads containing cell-specific collections of proteins, lipids and genetic material that are transported to other cells where they alter function and physiology^29^.

We thus hypothesized that p53 is accumulated in the cytoplasm and is stored in cytoplasmic microvesicles. Such a storage method allows cells to accumulate significant quantities of the protein without the visible manifestation of its biological activities. Microvesicles containing p53 are actively secreted by epithelial cells into the intercellular spaces and corneal tear film. They re-captured by other epithelial cells and play role in corneal anti-cancer defense.

At present, the role of p53 containing microvesicles can only be speculated. The study to clarify the significance of the factors described above is now underway.

## Supplementary information

Supplementary figure1.

## Data Availability

The analyzed datasets generated during the study are available from the corresponding author on reasonable request.
