# Supplementary material for: Features of p53 protein distribution in the corneal epithelium and corneal tear film
Source: Sci Rep. 2020 Jun 22;10:10051. doi: 10.1038/s41598-020-67206-z (PMC7308352; doi:10.1038/s41598-020-67206-z)
Supplement: Supplementary file 1 — Supplementary figure1. [file 41598_2020_67206_MOESM1_ESM.pdf]

Features of p53 protein distribution in the corneal epithelium and corneal tear film

Yevgeny Tendler and Alexander Panshin

Western blot analysis of the normal rat ocular tissues;
Fig.S1

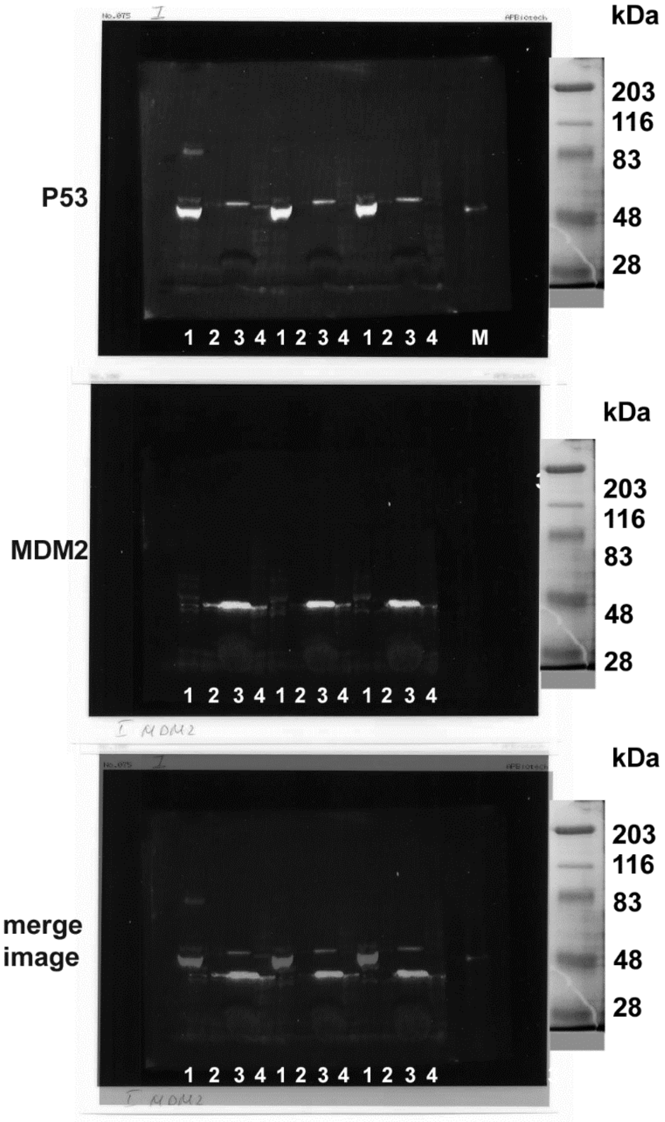

1, cornea; 2, iris; 3, lens; 4, retina; M, p53 positive control. Repeated designations 1, 2, 3, 4 are replicates of the same tissues. Western blot were subjected to western blot analysis using the MABs 248 and MDM2 (Clone SMP14) followed by HRP-conjugated anti-mouse IgG. See also, whole membrane image as supplementary Fig.S1.
